# Erector spinae plane block vs interscalene brachial plexus block for postoperative analgesia management in patients who underwent shoulder arthroscopy

**DOI:** 10.1186/s12871-022-01687-5

**Published:** 2022-05-12

**Authors:** Furkan Kapukaya, Mursel Ekinci, Bahadir Ciftci, Yunus Oktay Atalay, Birzat Emre Gölboyu, Ersin Kuyucu, Yavuz Demiraran

**Affiliations:** 1 Department of Anesthesiology and Reanimation, School of Medicine, Istanbul Medipol University, Istanbul, Turkey; 2 Department of Anesthesiology and Reanimation, Bursa City Hospital, Bursa, Turkey; 3 Department of Anesthesiology and Reanimation, School of Medicine, Katip Çelebi University, Izmir, Turkey; 4 Department of Orthopedics and Traumatology, Medical Park Bahçelievler Hospital, Istanbul, Turkey

**Keywords:** Erector spinae plane block, Interscalene brachial plexus block, Postoperative analgesia, Shoulder arthroscopy

## Abstract

**Background:**

Interscalene brachial plexus block (ISB) is the gold standard method used for postoperative analgesia after arthroscopic shoulder surgery. Ultrasound guided erector spinae plane block (ESPB) is an interfascial plane block. The aim of this study is to compare the analgesic efficacy of ESPB and ISB after shoulder arthroscopy. The primary outcome is the comparison of the perioperative and postoperative opioid consumptions.

**Methods:**

Sixty patients with ASA score I-II planned for arthroscopic shoulder surgery were included in the study. ESPB was planned in Group ESPB (*n* = 30), and ISB was planned in Group ISB (*n* = 30). Intravenous fentanyl patient-controlled analgesia was administered to both groups in the postoperative period. Intraoperative and postoperative opioid and analgesic consumption of both groups, side effects and complications related to opioid use, postoperative pain scores and rescue analgesic use were recorded in the first 48 h postoperatively.

**Results:**

Pain scores were significantly higher in the ESPB group in the first 4 h postoperatively than in the ISB group (*p* < 0.05). The total fentanyl consumption and number of patients using rescue analgesics in the postoperative period were significantly higher in the ESPB group (*p* < 0.05). The incidence of nausea in the postoperative period was significantly higher in the ESPB group (*p* < 0.05).

**Conclusions:**

In our study, it was seen that ISB provided more effective analgesia management compared to ESPB in patients underwent shoulder arthroscopy surgery.

## Introduction

Postoperative pain is a serious condition following shoulder arthroscopy. It causes the patient discomfort, negatively affecting the functional result of the surgery and preventing rehabilitation in the early postoperative period [1]. Various methods are used for postoperative pain management. Intravenous opioid agents are among them, but they may cause undesirable side effects, such as respiratory depression, sedation, constipation, allergic reaction, nausea, and vomiting [2]. Thus, alternative techniques are preferred.

Nowadays, several ultrasound (US)-guided regional anesthesia methods are used for postoperative analgesia. Regional techniques such as interscalene and supraclavicular blocks are usually preferred for shoulder analgesia. Interscalene brachial plexus block (ISB) is the gold standard technique in this area [3, 4]. Erector spinae plane block (ESPB) is a fascial plane block [5]. It has rapidly become popular following its first description in 2016. Shoulder analgesia is a novel usage area for ESPB [6]. There have been some case reports about its use in chronic and acute postoperative shoulder pain [7–9], but the studies about its efficacy in shoulder analgesia are limited. According to our detailed literature research, there has been only one randomized clinical trial (RCT) about the postoperative analgesic efficacy of high thoracic ESPB after shoulder surgery [10], and there has been no RCT in the literature that compares ESPB and ISB following shoulder arthroscopy [11]. In the study reported herein, we aimed to compare the effectiveness of ESPB and ISB for postoperative analgesia management in patients who underwent shoulder arthroscopy. Our hypothesis was ISB to be superior to ESB block prior to the commencement of the trial.

## Methods

**Fig. 1:**
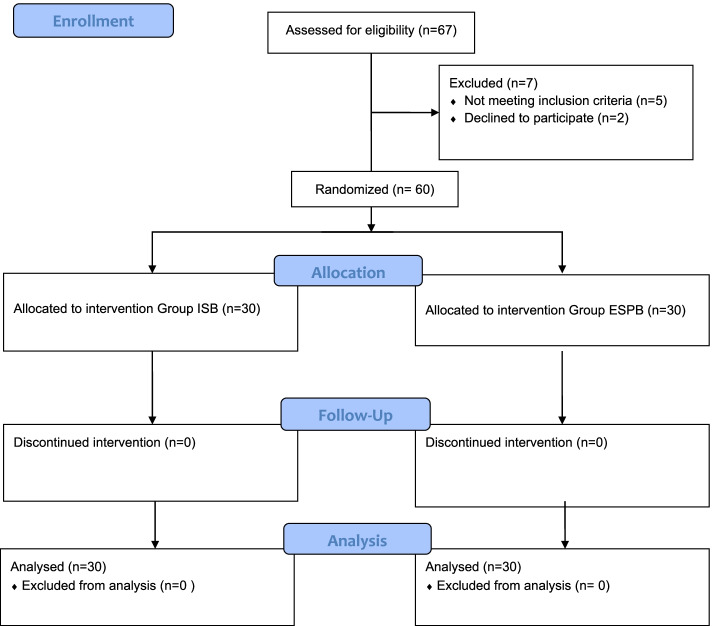
CONSORT flow diagram of the study

This randomized prospective study was approved by the Istanbul Medipol University Ethics and Research Committee (29.08.2019, Decision No. 26). After approval, the trial was recorded at ClinicalTrials.gov (NCT04083287) (10/09/2019). The study procedure was explained to the patients. After verbal explanation, written informed consent was obtained from the participants. The Consolidated Standards of Reporting Trials (CONSORT) flow diagram was used to report present study (Fig. 1). The study was conducted at Medipol Mega Hospital Complex from April 2020 to May 2021. All methods were carried out in accordance with relevant guidelines and regulations.

### Inclusion criteria

A total of 60 patients were included in the study. They were aged 18–65 years, were classified as American Society of Anesthesiologists (ASA) I or II, and were scheduled for elective unilateral shoulder arthroscopy. All the patients who were included in the study were informed about the ESPB and ISB procedures during their preoperative anesthesia visit. Their demographic data, such as their age, height, weight, gender, comorbidity, and ASA score, were recorded preoperatively.

### Exclusion criteria

Patients who had one or more of the following conditions were excluded from the study: previous coagulation or bleeding disorder, receiving anticoagulant therapy, allergy/sensitivity to local anesthetics (LAs) and/or opioids, infection in the procedure area (ESPB and ISB sides, corresponding to the T2 level and the neck, respectively), pregnancy or suspected pregnancy, breastfeeding mother, and refusal to undergo the procedure.

### Randomization

Sixty patients were randomized by a computer-generated algorithm from a computerized randomization program. The study assignment was sealed in opaque envelopes by pain nurse anesthetist. The envelopes were numbered between the numbers 1 and 60. Before arrival to the preoperative regional room, the anesthesiologist responsible for administering the block from the researcher anesthesia team opened the envelope. The group allocation was either a single-shot ISB (*n* = 30) or ESPB (*n* = 30). Only the pain nurse anesthetist who also evaluated the postoperative outcomes was blinded to the study.

### Block procedures

The ESPB and ISB procedures were performed in the preoperative regional room, followed by electrocardiography, peripheral oxygen saturation (SpO2), and non-invasive blood pressure monitoring. A 20 gauge intravenous (IV) cannula was placed in each patient, and 4 mL/kg 0.9% NaCl infusion was started in the room. The patients were sedated with 2 mg IV midazolam before the procedures. Then LA infiltration was applied to the block procedure area with 2% lidocaine. All the blocks were performed via US (Vivid Q) 30 min before the induction of general anesthesia. A high-frequency linear US probe (11–12 MHz, Vivid Q, Ge Healthcare, USA) was covered with a sterile sheath under aseptic conditions, and a 50 mm block needle (Braun 360°) was used.

### ESPB procedure

**Fig. 2:**
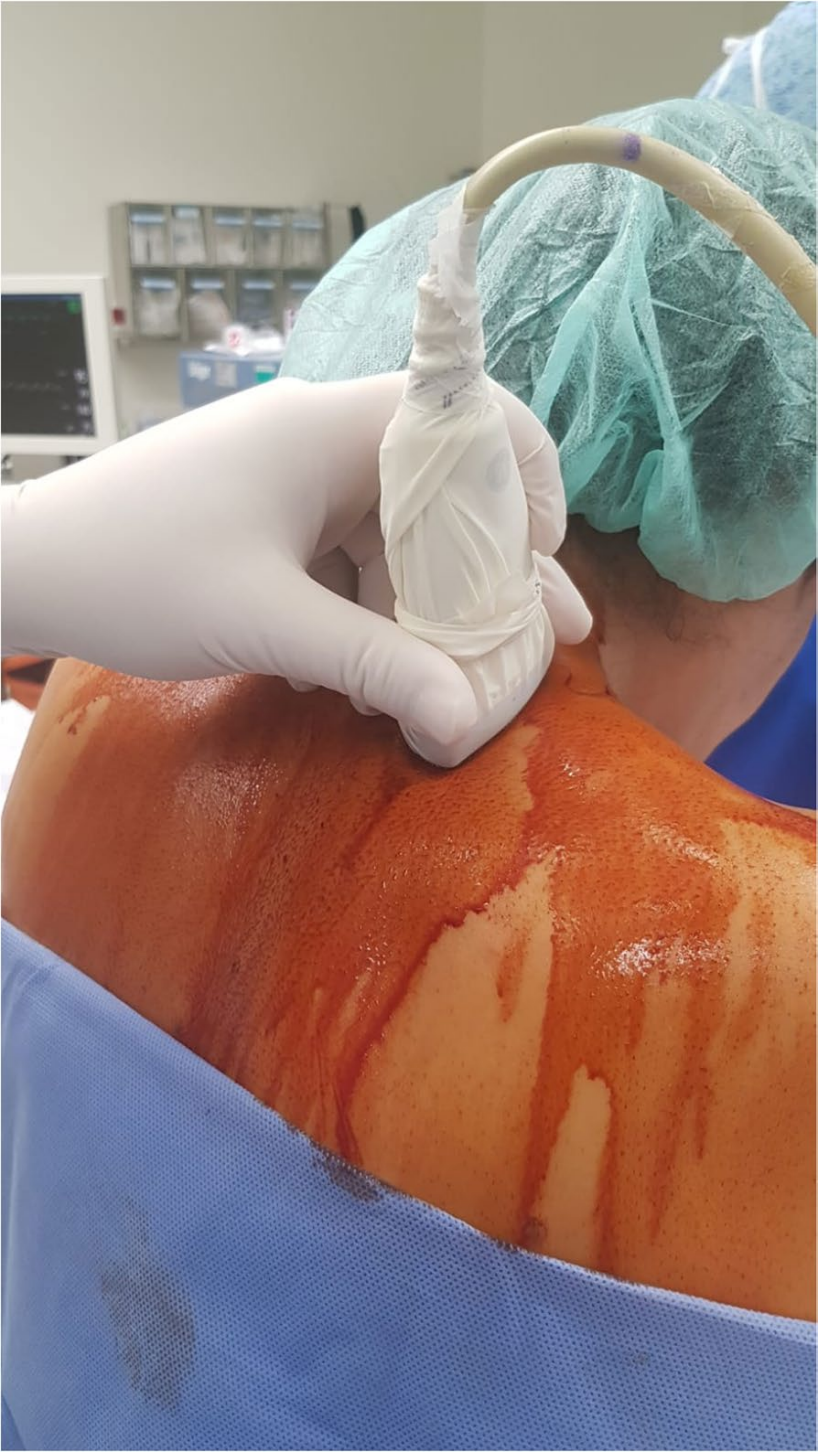
Patient position under aseptic conditions during T2 ESPB performing

ESPB was performed by the researcher anesthesia team in the sitting position before induction, under aseptic conditions, using a high-frequency linear US probe. The US probe was placed longitudinally 2–3 cm lateral to the T2 spinous process [7–9, 12] (Fig. 2). The erector spinae muscle was seen here. Using the “in plane” technique, the block needle was inserted into the skin in the caudo-cranial direction. When the trapezius, rhomboid, and erector spinae muscles were passed and the needle touched the transverse process (approximately 3 cm in depth), 5 mL saline was injected into the area between the erector spinae muscle fascia and the vertebral transverse process. Thus, the block site was confirmed. After confirmation, 30 mL of 0.25% bupivacaine was administered.

### ISB procedure

ISB was applied while the patient was in the supine position. The probe was placed transversely at the level of the cricoid cartilage, and when the artery was identified, the probe was moved slightly laterally. After the visualization of the brachial plexus between the scalene muscles, the block needle was advanced from the lateral to the medial direction with the “in plane” technique, and the block location was confirmed by applying 5 mL saline injection. After the block location was confirmed, 30 mL of 0.25% bupivacaine was administered.

After the success of the blocks was tested using a cold test with an ice pack, the patients were taken to the operating room. The ESPB and ISB were considered successful due to the presence of anesthesia in the corresponding dermatomal area (C5-T1).

### General anesthesia

Intubation was performed in both groups by administering 2–2.5 mg/kg propofol (Lipuro, Braun), 1–1.5 μg/kg fentanyl (Talinat, VEM), and 0.6 mg/kg rocuronium (Esmeron, Alessandroorsini) intravenously to the patients in both groups. Anesthesia was maintained with sevoflurane (Sevorane, Abbott) in a mixture of O_2_/air in a 50/50% concentration and remifentanil (Ultiva, VLD) infusion (0.01–0.1 μg/kg/min). The mechanical-ventilator settings were adjusted to provide a 6–8 mL/kg tidal volume and a 30–35 mmHg end-tidal CO_2_ level. If the pulse or mean arterial blood pressure increased by 20% from the preoperative baseline value, 25 μg bolus fentanyl and 0.1 mg/kg rocuronium were administered intravenously. Arthroscopic shoulder surgery was performed by the same surgical team using the same technique, in the beach chair position. To prevent nausea and vomiting, 4 mg ondansetron was administered intravenously. After extubation, the patients with sufficient spontaneous respiration were taken to the recovery room.

### Analgesia protocol

We used multimodal analgesia protocol in our study. Preemptive 400 mg ibuprofen was administered intravenously to all the patients before the surgical procedure. A dose of 100 mg tramadol was administered intravenously 30 min before the end of the surgical procedure. In the postoperative period, 400 mg ibuprofen was administered intravenously every 8 h. Patient-controlled analgesia containing 10 μg/mL fentanyl was provided intravenously to the patients in all the groups (10 μg bolus without infusion dose, 10 min lock-in time protocol). For rescue analgesia, we used 0.5 mg/kg IV meperidine.

### Postoperative analgesia assessment and outcomes

In the postoperative period, the patients were evaluated by another pain nurse who did not know the composition of the groups. The postoperative pain was assessed using the visual analogue scale (VAS) score (0 = no pain; 10 = the most severe pain felt). The VAS scores were recorded at rest (static) and at mobilization (dynamic) at the 1st, 2nd, 4th, 8th, 16th, 24th, and 48th h. If the VAS score was ≥ 4, 0.5 mg/kg IV meperidine was used as rescue analgesia. Perioperative and postoperative opioid consumption, adverse effects (e.g., nausea, vomiting, itching, complications) that may occur due to blocks such as respiratory failure, and hematoma were recorded.

The primary outcome was the result of the comparison of the perioperative and postoperative opioid consumptions. The secondary outcomes were the evaluation of the postoperative pain scores, the complications related to blocks, and the opioid-related side effects.

### Statistical analysis and sample size calculation

The mean sample size was calculated using the G*Power 3 analysis program (Heinrich-Heine-Universitat Düsseldorf, Germany). A preliminary study was performed with 16 patients (ESPB group = 8; ISB group = 8). The power analysis was based on the mean fentanyl consumption, which was the primary outcome of the study. The mean fentanyl consumption was 62.85 μg (± 39.03) in the ISB group and 91.4 μg (± 41.4) in the ESPB group. We considered 50% reduction in fentanyl consumption clinically meaningful and important. The sample size was calculated at 80% power and at a 5% significance level, and it was determined that at least 28 patients per group were required to obtain a statistically significant value. Therefore, we included 30 patients in each group to prevent possible dropouts.

Statistical analyses were performed using IBM SPSS Statistics for Windows (Version 22.0; IBM Corp., Armonk, NY, USA). The Kolmogorov–Smirnov test was used to analyze the data distribution, and the Pearson chi square test was used to compare the categorical data (gender, ASA status, rescue analgesic usage, incidence of adverse effect) between groups. Student’s t-test was used to control for differences between the groups at the 5% significance level for the normally distributed continuous variables (demographic datas and duration times of surgery and anesthesia, postoperative fentanyl consumption, introperative remifentanyl consumption and of rescue analgesia consumption, VAS scores). The descriptive statistics were expressed as mean ± standard deviation. In our study, the statistical significance threshold was *p* < 0.05.

## Results

The prospective study reported herein included a total of 60 participants (30 in each of the two groups). There were no significant differences between the groups in terms of demographic data, ASA classification, anesthesia duration, and length of surgery (*p* > 0.05) (Table 1).

**Table 1:** Comparison of demographic datas and duration times of surgery and anesthesia between group ISB and ESPB

	**Group ISB** **(n:30)**	**Group ESPB** **(n:30)**	**95% CI**
Gender (M/F)	17 / 13	15 / 15	
Age (years)	45.07 ± 14.72	47.03 ± 13.3	-9.21 to 5.28
Weight (kg)	79.17 ± 14.11	80.8 ± 11.18	-8.21 to 4.94
Height (cm)	170.3 ± 9.95	168 ± 8.5	-2.63 to 6.96
ASA I/II	16 / 14	11 / 19	
Duration of surgery (min)	100.1 ± 46.79	98.46 ± 33.76	-19.38 to 22.78
Duration of anesthesia (min)	150.4 ± 55.32	153.4 ± 32.11	-26.41 to 30.34

Values are expressed mean ± standart deviation or number

*kg* kilogram, cm; centimeter, *M* male, *F* female, min; minutes, *ASA* American Society of Anesthesiologist

There was no significant difference between the groups in the first 8, 8–16, 16–24, and 24–48 h post-operation in terms of fentanyl consumption (*p* > 0.05). However, the postoperative total fentanyl consumption was significantly higher in the ESPB group than ISB group (81 ± 47.54 mcg, 56 ± 35.38 mcg; respectively) (95% CI; -46.99 to 3.67) (*p* = 0.023). The number of patients that needed rescue analgesia was higher in the ESPB group than ISB group (15 / 15, 6 / 24; respectively) (*p* = 0.015). The rescue analgesic dose was higher in the ESPB group than ISB group (25.33 ± 30.31 mg, 8.33 ± 17.23 mg; respectively) (95% CI; -29.74 to 4.25) (*p* = 0.010). There was no significant difference between ESPB and ISB groups in terms of intraoperative remifentanil consumption (858.2 ± 486.48 mcg, 882.67 ± 583.91 mcg; respectively) (95% CI; -253.29 to 302.22) (*p* = 0.861) (Table 2).

**Table 2:** Comparison of the postoperative fentanyl consumption, introperative remifentanyl consumption and the use of rescue analgesia (meperidin) between group ISB and ESPB

	**Group ISB** **(n:30)**	**Group ESPB** **(n:30)**	**P**	**95% CI**
0-8^th^ hours (mcg)	14.66 ± 16.55	23.33 ± 24.11	0.110	-19.35 to 2.02
8-16^th^ hours (mcg)	23.33 ± 20.39	26.66 ± 24.11	0.550	-14.42 to 7.76
16-24^th^ hours (mcg)	12 ± 13.49	24.6 ± 22.18	0.092	-27.44 to 2.11
24-48^th^ hours (mcg)	6 ± 10.69	24.66 ± 17.2	0.371	-10.73 to 4.07
Rescue analgesic using (yes/no)	6 / 24	15 / 15	**0.015 **^**β**^	
Rescue analgesic dose (mg)	8.33 ± 17.23	25.33 ± 30.31	**0.010 **^**α**^	-29.74 to 4.25
İntraoperative remifentanyl consumption (mcg)	882.67 ± 583.91	858.2 ± 486.48	0.861	-253.29 to 302.22
Total Fentanyl consumption (mcg)	56 ± 35.38	81 ± 47.54	**0.023 **^**α**^	-46.99 to 3.67

Values are expressed mean ± standart deviation or numbers

*mcg* microgram, *mg* milligram

^α^*p* < 0.05 Independent Student’s T test between groups

^β^*p* < 0.05 Chi-square test between groups

**Fig. 3:**
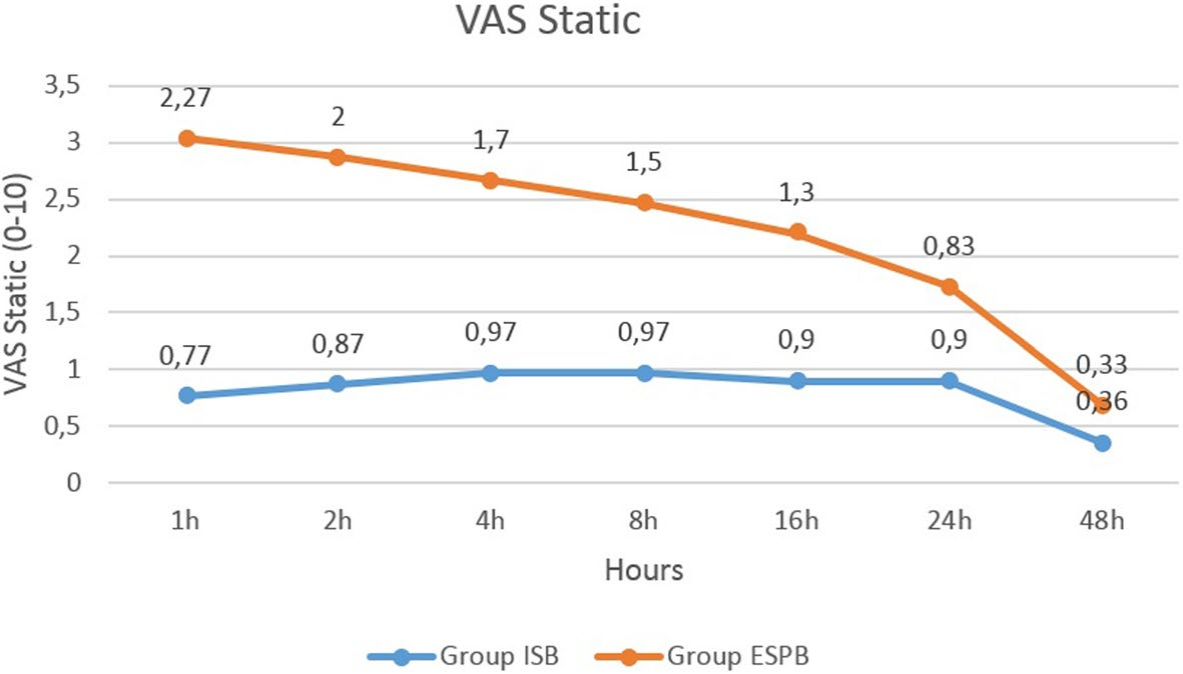
Comparison of the static visual analogue scale scores between group ISB and ESPB

**Fig. 4:**
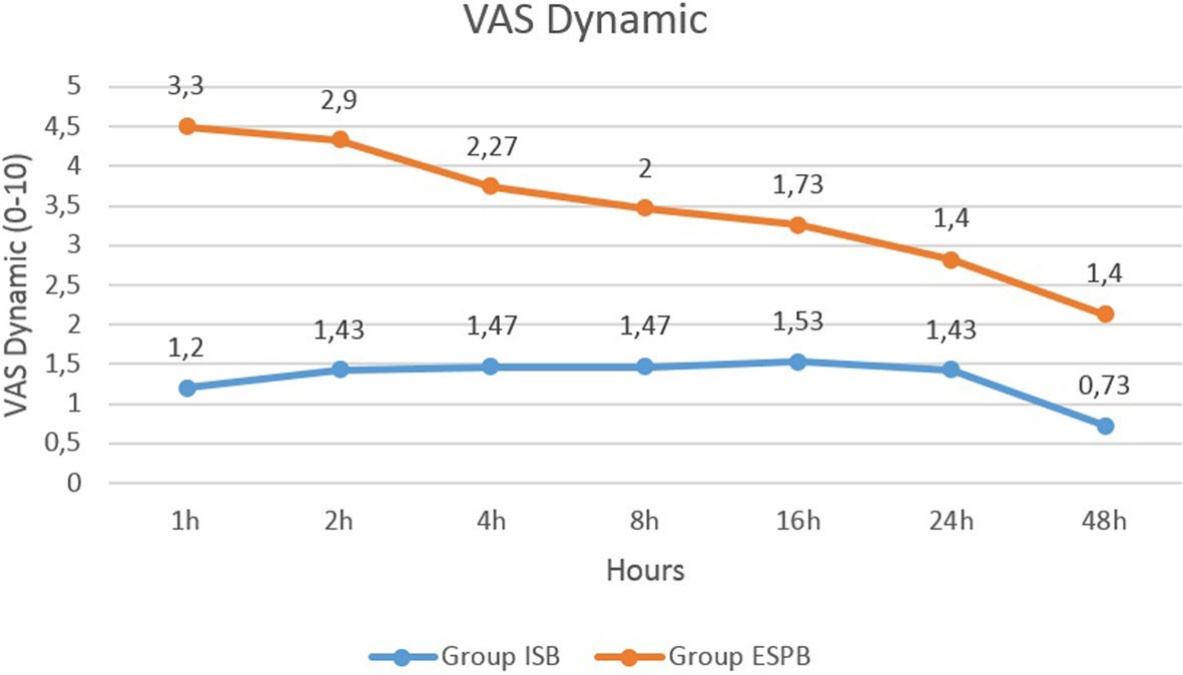
Comparison of the dynamic visual analogue scale scores between group ISB and ESPB

In terms of VAS, in the ESPB group, both the dynamic and static VAS scores 1 (3.30 ± 2.07, 2.27 ± 1.5 respectively), 2 (2.9 ± 1.58, 2 ± 1.14 respectively), and 4 h (2.27 ± 1.20, 1.7 ± 1.05 respectively) post-operation were significantly higher than those in the ISB group (1.2 ± 1.73, 0.77 ± 1.25; 1.43 ± 2.9, 0.87 ± 1.27; 1.47 ± 1.45, 0.97 ± 1.06; dynamic and static VAS respectively) (*p* < 0.05). There was no significant difference between the VAS scores in both groups at the other time intervals (*p* > 0.05) (Fig. 3 and 4).

As for the side effects, nausea, vomiting, and itching were observed in both groups. The incidence of nausea in the postoperative period was significantly higher in the ESPB group than in the ISB group (10 / 20 patients, 3/27 patients; respectively) (*p* = 0.028). There was no significant difference between the two groups in terms of vomiting and itching (*p* > 0.05) (Table 3). There were no other side effects or complications related to blocks.

**Table 3:** The Comparison of incidence of adverse effects between group ISB and ESPB

	**Group ISB** **(n:30)**	**Group ESPB** **(n:30)**	**P**
Nausea (yes/no)	3 / 27	10 / 20	**0.028**^**α**^
Vomiting (yes/no)	2 / 28	6 / 24	0.129
Itching (yes/no)	1 / 29	2 / 28	0.554

*p* < 0.05 Chi-square test between groups

## Discussion

Our study was designed to compare the efficacy of ESPB and ISB following arthroscopic shoulder surgery. The results of our study showed that ISB provided lower opioid consumption than ESPB 48 h post-operation. The VAS scores 1, 2, and 4 h post-operation were significantly lower in the ISB group. There was no difference between the two groups’ VAS scores 8, 16, 24, and 48 h post-operation. The use of rescue analgesia in the postoperative period was significantly lower in the ISB group. There was no difference in intraoperative opioid consumption.

Shoulder arthroscopy is a very common procedure and is usually performed as a daily surgery [1]. The level of pain in the first 24 and 48 h after arthroscopic shoulder surgery is generally similar to the pain after open surgery, and 30% of the patients suffer severe pain on the first day post-operation [12]. Complications such as insufficient postoperative rehabilitation and prolongation of hospital stay may occur due to pain [1]. Therefore, several methods are used for postoperative analgesia in patients undergoing arthroscopic shoulder surgery. For years, different regional methods have been used for postoperative analgesia in shoulder surgery. These include ISBs such as local infiltration, suprascapular nerve block with or without axillary nerve block, superficial cervical plexus block, and supraclavicular block [3, 4].

The brachial plexus branches originate from the C5–C8 levels and innervate a large part of the shoulder joint. The anterior part of the shoulder joint is innervated by the axillary, lateral pectoral, and subscapular nerves, and these nerves are branches of the posterior and medial cords of the brachial plexus [13]. The suprascapular nerve (C5–C6) originates from the upper trunk of the brachial plexus, and together with the axillary nerve, supplies the sensory innervation of the posterior and superior 70% of the shoulder joint [14]. The innervation of the shoulder capsule is provided not by the brachial plexus but by the supraclavicular nerves, which are branches of the superficial cervical plexus (C3–C4) [15]. ISB has been accepted as the gold standard for postoperative analgesia in patients undergoing shoulder surgery. It also spreads to the supraclavicular nerve and the brachial plexus [16]. In previous studies, a single dose of ISB has been shown to have clinically significant analgesic effects lasting 12 [17, 18] or even 24 h [19, 20]. Our results showed that the postoperative static and dynamic VAS scores 8 and 48 h post-operation were similar in the ISB and ESPB groups. Due to this similarity, ESPB may be a good alternative to ISB, especially in patients that have pulmonary pathology. One of the most common side effects of ISB is an ipsilateral phrenic-nerve block, ESPB may be preferred for patients with limited vital capacity. In addition; in obese patients with short thick necks, it may be difficult to visualize the sonographic anatomy of ISB. In such cases, ESPB may be preferred instead of ISB.

ESPB is a regional technique for analgesia management after a shoulder operation. It may also be used for chronic-pain management and upper-extremity operations [6, 8, 21]. It has been shown in cadaver and radiological imaging studies that ESPB spreads to the paravertebral area via the connective tissue and ligaments [22–25]. ESPB is in fact a fascia block, but it was defined as a paraspinal block by Chin et al. [26]. The local anesthetic spreads to the C4–C8 levels during T2 ESPB [8]. As a result, the branches of the brachial plexus, musculocutaneous, axillary, median, radial, and ulnar nerves may be affected. Although the shoulder joint capsule is innervated from the superior cervical plexus (C3–C4), a sensory blockade may be provided in the shoulder joint with ESPB [7, 8]. Forero et al. showed that the local anesthetic spreads to the C3–C7 levels during T2 ESPB in computed tomography images, and is effective for the analgesia management of chronic pain [8]. Ciftci et al. performed ESPB in patients who underwent arthroscopic shoulder surgery and showed that ESPB provided lower VAS scores even 48 h post-operation compared to the “sham” group [10]. Shanthanna et al. performed a randomized controlled trial with double-dummy design comparing ESPB with peri-articular injection (PAI) [27]. They reported that ESPB was not superior to PAI in terms of pain control following major arthroscopic shoulder repair surgery. Our present trial is another study about the efficacy of ESPB for shoulder analgesia. Our results show that ISB is superior to ESPB in terms of analgesia management after shoulder surgery. Since there is a limited number of study about the analgesic efficacy of ESPB in this area, further studies are needed for success of high thoracic ESPB for shoulder surgery. Moreover, ISB (differently from ESPB) needs a multimodal monitoring in order to ensure a high level of safety in order to avoid needle-nerve contact which may cause a potential nerve damage. In a prospective study, Pascarella et al. performed triple monitoring during ISB in terms of intraneural injection [28]. In the study, they used a combination of ultrasound US, nerve stimulation, and opening injection pressure during ISB for shoulder surgery. They reported that triple monitoring was useful and feasible while performing ISB for arthroscopic shoulder surgery. To compare the safety profile of ISB and ESPB further studies are needed.

### Limitations

Our study had some limitations. We used a concentration of 0.25% bupivacaine in a 30 mL volume. More studies may be performed with different LA concentrations. Radiological studies may be required to demonstrate high-volume LA spread. We applied ESPB with a single injection, but a continuous infusion block catheter can be used in the postoperative period. We performed ESPB at T2, not cervical. Maybe the results would be different with cervical ESPB. For this reason, future studies are expected, perhaps with a higher approach (cervical) to ESPB in order to confirm or not our findings. We did not evaluate the effect of ESPB on diaphragm function. Objective pulmonary function (with spirometry or US visualization of diaphragm movement) would be best for our study. Lastly, we performed ESPB as a single injection, and that multiple ESPB injections were not explored.

Further combination of fascial blocks have to be explored before ruling out ESP block from shoulder surgery as such combinations have been shown to be promising in other settings [29]. As a limitation, we performed ESPB alone, not in combination with another fascial plane blocks. The results would be different with combinations vs ISB in future studies.

## Conclusion

According to the results of our study, ISB provided lower postoperative opioid consumption and pain scores. ISB provides more effective pain control than ESPB following arthroscopic shoulder surgery.

## Data Availability

The datasets generated and/or analysed during the current study are not publicly available, but are available from the corresponding author on reasonable request.

## Abbreviations

ASA: American Society of Anesthesiologists
CONSORT: The Consolidated Standards of Reporting Trials
ESPB: Erector spinae plane block
ISB: Interscalene brachial plexus block
LA: Local anesthetics
PCA: Patient-controlled analgesia
RCT: Randomized clinical trial
US: Ultrasound
VAS: Visual analogue scale
